# Curative immunotherapy-based strategies for non-metastatic non-small cell lung cancer

**DOI:** 10.37349/etat.2024.00281

**Published:** 2024-12-06

**Authors:** Justin J. Kuhlman, Shenduo Li, Rami Manochakian, Yanyan Lou, Yujie Zhao

**Affiliations:** IRCCS Istituto Romagnolo per lo Studio dei Tumori (IRST) “Dino Amadori”, Italy; Division of Hematology and Medical Oncology, Mayo Clinic, Jacksonville, FL 32224, US

**Keywords:** Non-small cell lung cancer, immunotherapy, molecularly targeted therapy, immune checkpoint inhibitor

## Abstract

The emergence of immunotherapy has ushered in a new era in the management of non-small cell lung cancer (NSCLC). Various immune check point inhibitors have demonstrated significant benefit in the management of locally advanced NSCLC that are treated with either surgery or concurrent chemoradiation. We provide a comprehensive and up-to-date review of data from key studies, discuss the challenging clinical issue regarding the timing and duration of immunotherapy in patients undergoing surgery, and highlight the unmet needs and future directions of immunotherapy in NSCLC.

## Introduction

Lung cancer is the second most common cancer across the globe and represents the leading cause of death among all cancer subtypes in both men and women [1]. Non-small cell lung cancer (NSCLC) composes the largest majority (~85%) of lung cancer cases and includes the histological subtypes of adenocarcinoma (50%), squamous cell carcinoma (30%), and large cell carcinoma (5%) [2, 3]. Approximately 44.8% of NSCLC patients present with stage IV metastatic disease at initial diagnosis, while 22.3% and 28.1% of patients had either regional lymph node involvement or localized disease only, respectively, according to data spanning from 2017 to 2021 [4].

**Figure 1:**
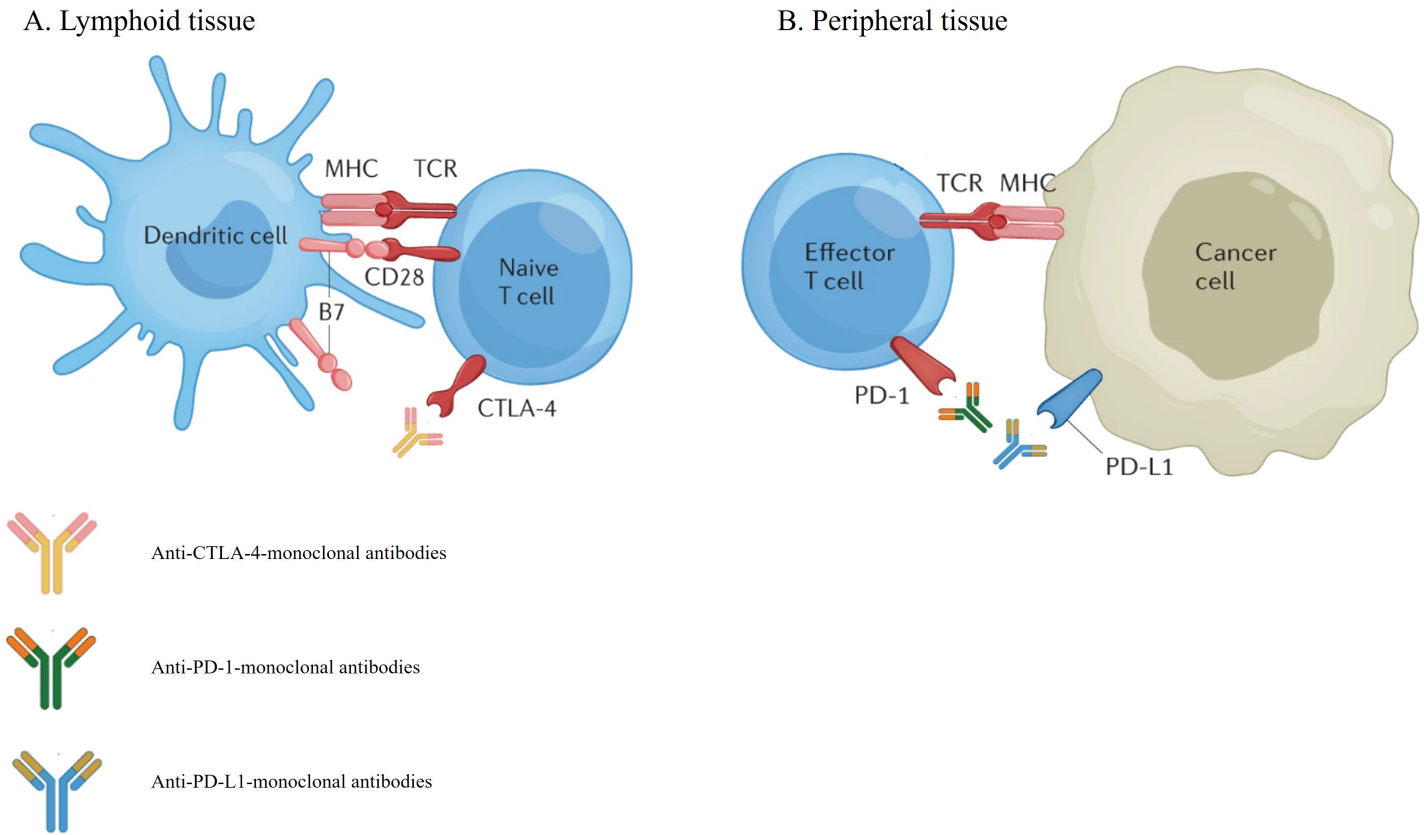
Mechanisms of action of immune-checkpoint inhibitors targeting CTLA-4 (A) and PD-(L)1 (B) [10]. CTLA-4: cytotoxic T lymphocyte antigen 4; PD-1: programmed death 1; PD-L1: programmed death 1 ligand *Note.* Adapted with permission from “Immune-checkpoint inhibitor use in patients with cancer and pre-existing autoimmune diseases” by Tison A, Garaud S, Chiche L, Cornec D, Kostine M. Nat Rev Rheumatol. 2022;18:641–56 (https://www.nature.com/articles/s41584-022-00841-0). Copyright © 2022, Springer Nature Limited

**Figure 2:**
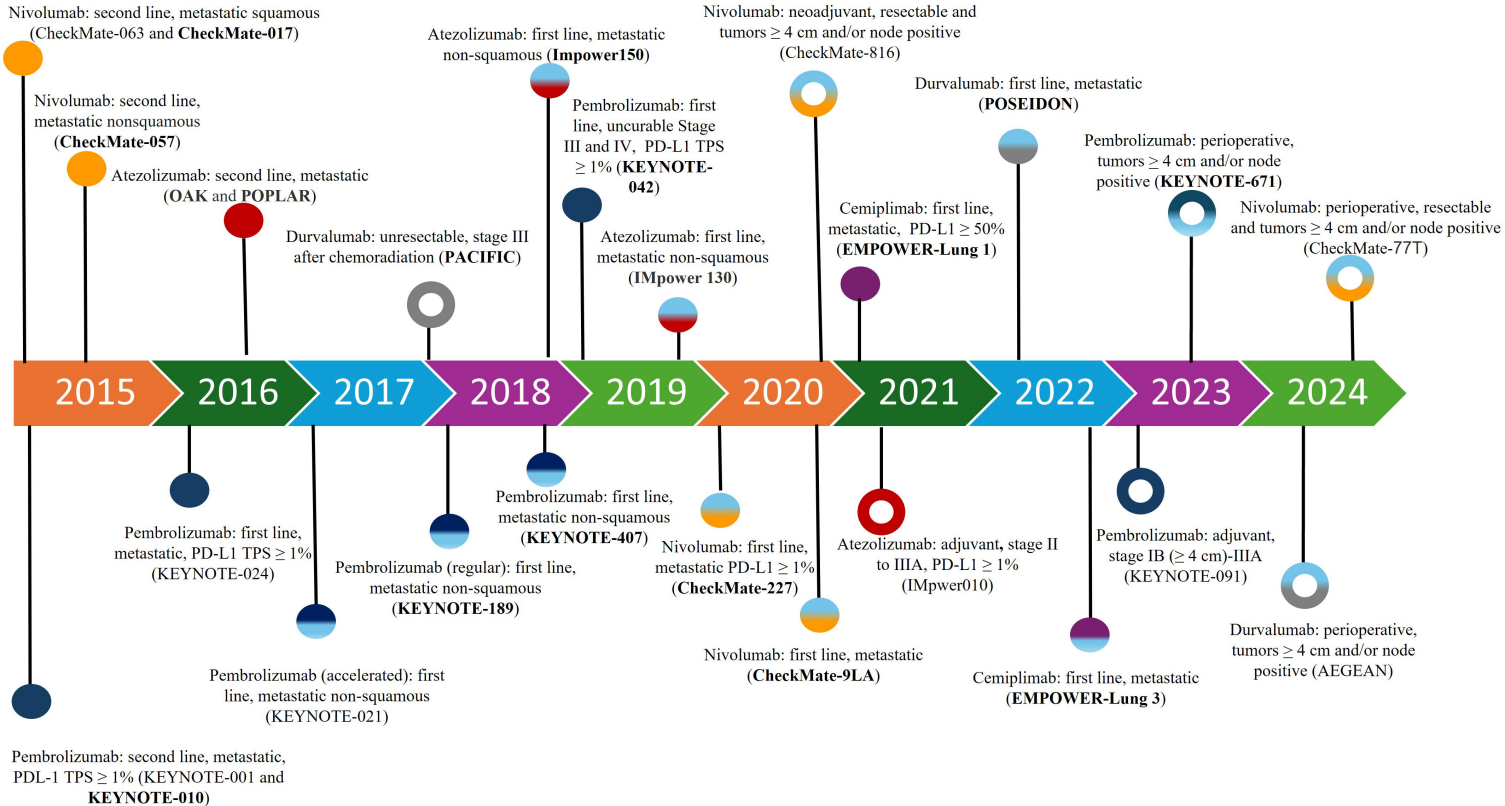
FDA approval timeline of immune checkpoint inhibitors in NSCLC. Solid circle: stage IV or stage III disease that cannot be treated with surgery or radiation. Open circle: stage II–III disease. Unicolor circle: single agent treatment. Multicolor circle: immune checkpoint inhibitor in combination with chemotherapy with or without monoclonal antibody targeting vascular endothelial growth factor or CTLA-4. Clinical trials with OS as a primary endpoint are in bold. FDA: Food and Drug Administration; NSCLC: non-small cell lung cancer; OS: overall survival

The mainstay of the management of patients with metastatic NSCLC is systemic therapy. For patients with disease harboring actionable molecular alterations, targeted therapies are often the preferred treatment, whereas for patients without suitable targeted therapy, while cytotoxic chemotherapy remains an important option, monoclonal antibody-based immunotherapy agents have demonstrated significant survival benefits and have become a crucial component in the management of NSCLC [5, 6]. These agents, known as immune checkpoint inhibitors (ICI), can exert robust anti-tumor activity by blocking checkpoint proteins from binding with their partner proteins, thereby “unleashing” T-cell responses against cancer cells [6]. The two major T cell immune checkpoint molecules targeted by immunotherapy in NSCLC are cytotoxic T lymphocyte antigen 4 (CTLA-4) and PD-(L)1. These molecules negatively regulate T-cell immune function at different stages and anatomic locations during immune responses and play essential roles in maintaining immune homeostasis and preventing autoimmunity. CTLA-4 induces T-cell anergy at the initial stage of naive T-cell activation by outcompeting the co-stimulatory receptor CD28 from binding CD80 (B7-1) and CD86 (B7-0 or B7-2), typically in lymph nodes, whereas the programmed death 1 (PD-1) pathway is active during simultaneous T cell activation during the later stages of immune response, primarily in peripheral tissues (Figure 1) [7–9]. Over the past decade, multiple monoclonal antibodies, blocking either PD-(L)1 or CTLA-4, have been evaluated in many randomized phase III clinical trials and were able to show significant survival benefit with favorable toxicity profiles in patients with NSCLC. Many of these regimens subsequently gained U.S. Food and Drug Administration (FDA) approval in uncurable stage III and IV NSCLC (Figure 2).

NSCLC confined to the primary site and regional lymph node(s) is typically treated with a curative-intent with either surgery or radiation. For stage II or stage III diseases, platinum-based combination chemotherapy is typically offered as adjuvant therapy following surgery or concurrently with radiation to improve the outcome. However, even with the combination of chemotherapy and radiotherapy, the median progression-free survival (PFS) and 5-year overall survival (OS) in patients with unresectable stage III NSCLC remained dismal at 8 months and 15%, respectively. Various efforts, including incorporation of induction chemotherapy or consolidative chemotherapy, targeted therapy, antigen-specific cancer immunotherapy, and angiogenic inhibitors, have been made to improve the outcomes in this patient population, but none were found to be practice-changing [11–18]. Moreover, in patients who undergo surgery as the primary treatment, neoadjuvant or adjuvant platinum-based combination chemotherapy was only able to improve OS by around 5% in stage II–III diseases [19–21]. It was not until the recent decade when ICI emerged as a promising component in the multimodal treatment paradigm of locally advanced disease. The significant survival benefit associated with ICI in the metastatic setting suggested the possibility of elimination of NSCLC micrometastases by antitumor T cells and triggered the interest in investigating the potential role of ICI in the curative-intent approach. A series of phase III studies were subsequently carried out to evaluate ICI in the adjuvant, neoadjuvant, and peri-operative treatment settings in patients undergoing surgery, and as a consolidative treatment following concurrent chemoradiation (CRT) in patients with stage III NSLCL. The positive findings in these trials have led to the approval of ICI in patients undergoing curative treatment with either surgery or CRT.

In this review, we summarize recent advancements in the management of locally advanced disease and highlight the role of immunotherapy and its various combinations. We offer our opinion on different treatment strategies in resectable NSCLC based on available evidence. Finally, we discuss the limitations of the current landscape and provide insights towards future directions.

## The role for immunotherapy in resectable disease

### Immunotherapy strategies in the neoadjuvant setting

Signals of therapeutic efficacy from the use of immunotherapy alone in the neoadjuvant setting in resectable NSCLC were initially demonstrated in a pilot study involving the administration of two doses of nivolumab, a PD-1 blocker, to 22 patients with stage I–IIIA (AJCC 7th edition staging) NSCLC [22]. Of the 21 tumors that were surgically resected, 20 were completely resected and 9 (45%) demonstrated a major pathological response. Another trial designed to investigate the possible additive value of neoadjuvant immunotherapy was the randomized phase II NEOSTAR trial [23]. In this study, the NEOSTAR trial assessed the rate of major pathological response with the PD-1 inhibitor, nivolumab, in combination with the CTLA-4 inhibitor, ipilimumab, compared with nivolumab alone in 44 patients with operable stage IA–IIIA NSCLC (AJCC 7th edition). Combination nivolumab and ipilimumab resulted in a major pathological response rate of 38% compared to 22% in those who received nivolumab alone in the intention-to-treat (ITT) population. The pathological complete response (pCR) rate among patients who underwent resection was 38% in the combination arm and 10% with nivolumab alone. Similarly, the subsequent LCMC3 phase 2 trial, although a single arm trial, demonstrated that two doses of neoadjuvant atezolizumab, a programmed death 1 ligand (PD-L1) inhibitor, resulted in a major pathologic response rate of 20% and a pCR rate of 7% in those with stage IB to IIIB disease (AJCC 8th edition) [24]. Based on these trials, and compared to historical standards, neoadjuvant immunotherapy alone appeared to be superior to induction chemotherapy which historically had been associated with a median pCR rate of only 4% [25]. In a phase 2 trial, neoadjuvant combination chemotherapy-ICI (chemo-ICI) with atezolizumab was investigated in patients with stage IB–IIIA (AJCC 7th edition) NSCLC as 4 cycles of atezolizumab, nab-paclitaxel, and carboplatin were given to 30 patients in this single arm study [26]. The major pathological response rate was 57%, providing further evidence towards the benefit of combining ICI with chemotherapy in the neoadjuvant setting.

The benefits witnessed in the previous trials were finally confirmed in the phase 3 CheckMate 816 trial, which randomized 358 patients with stage IB (≥ 4 cm)–IIIA (AJCC 7th edition) NSCLC without ALK translocations or epidermal growth factor receptor (EGFR) mutations to three cycles of nivolumab plus platinum-based chemotherapy or platinum-based chemotherapy alone [27]. The study met its primary endpoints with improved pCR (24% vs. 2%) and event free survival (EFS) (31.6 months vs. 20.8 months) in those who received chemo-ICI compared to the chemotherapy alone arm, respectively. In the subgroup analysis, when stratifying patients according to PD-L1 expression, there appeared to be a greater benefit in those who harbored a PD-L1 ≥ 1%, especially in those with a PD-L1 ≥ 50%. Additionally, circulating tumor DNA (ctDNA) was evaluated in 89 patients, and a higher ctDNA clearance rate was observed in those who received nivolumab. Clearance of ctDNA was also associated with longer EFS and a higher rate of pCR. In terms of treatment related toxicities, 40.9% and 43.8% of patients experienced grade 3 or 4 adverse events in the nivolumab treatment arm and placebo arm, respectively. The most common grade 3 or 4 treatment-related adverse events were related to neutropenia, which occurred in 8.5% of those who received nivolumab plus chemotherapy compared with 11.9% in those who received chemotherapy alone. Only 5.7% of patients discontinued treatment due to treatment-related toxicity in the nivolumab arm compared to 4% in the chemotherapy alone arm. Events accredited to immune mediated side effects occurred in 10.2% of patients receiving nivolumab and most frequently included rash (8.5%) and thyroid disorders (6.3%). Only 3.4% of all immune mediated events were grade 3 or higher. These events included rash, adrenal insufficiency, and hypophysitis. More recently, the updated four-year survival analysis suggested a trend in improved OS in the nivolumab plus chemotherapy arm compared against the chemotherapy alone arm [hazard ratio (HR) 0.71; 95% CI 0.47–1.07; *P* = 0.0451; median OS not reached in either arm], although the data remains insignificant [28]. Consequently, nivolumab in combination with chemotherapy has gained FDA approval as a neoadjuvant treatment option for adult patients with resectable NSCLC (tumors ≥ 4 cm or node positive) and is supported by current NCCN Clinical Practice Guidelines in Oncology [6]. It is also worth noting that patients with EGFR mutations or ALK rearrangements were excluded from enrollment. Excluding diseases harboring these genetic alterations, therefore, is essential prior to considering neoadjuvant platinum-based chemotherapy with nivolumab. In summary, NSCLC patients with tumors ≥ 4 cm and/or with node positive disease who are deemed to have resectable disease and who are without contraindications to immunotherapy should be assessed for the neoadjuvant chemo-ICI approach with nivolumab as one of the options. This approach should be avoided in patients with diseases harboring EGFR exon 19 deletions, EGFR exon 21 L858R mutations, or ALK fusions [29, 30].

### Immunotherapy strategies in the perioperative setting

A perioperative immunotherapy approach, that is, both neoadjuvant and adjuvant immunotherapy combined with chemotherapy, has also now been investigated and established as another treatment option for those with resectable disease. Theoretically, perioperative immunotherapy would not only be efficacious in the setting of increased antigen presentation and immunogenic stimulation from a larger tumor burden prior to surgery but can also eradicate residual micro metastases and maintain immunological defenses against recurrence after surgery with ongoing adjuvant treatment.

Combination chemo-ICI in the perioperative setting was explored in the single arm, phase 2, NADIM trial [31]. All patients in the trial had surgically resectable stage IIIA disease (AJCC 7th edition) and were given three cycles of carboplatin, paclitaxel, and nivolumab in the neoadjuvant setting, followed by adjuvant nivolumab monotherapy for 1 year after surgery. The rate of major pathological response was an impressive 83% with a pCR rate of 63% following neoadjuvant chemo-ICI administration. Additionally, there was a significant correlation between undetectable ctDNA levels and improved PFS and OS with HR of 0.26 and 0.04, respectively.

The landmark KEYNOTE 671 trial investigated the peri-operative immunotherapy approach. In this randomized, double blind, phase 3 trial, 797 patients with resectable stage II–IIIB NSCLC (AJCC 8th edition) were assigned to neoadjuvant pembrolizumab or placebo every 3 weeks, each of which was given with cisplatin-based chemotherapy for 4 cycles, followed by surgery and adjuvant pembrolizumab or placebo every 3 weeks for up to 13 cycles [32]. The study had dual primary endpoints of EFS and OS and pathological responses were included as secondary endpoints. Patients who received pembrolizumab demonstrated significantly improved median EFS (47.2 months vs. 18.3 months, HR 0.59; 95% CI, 0.48–0.72) and OS (not reached vs. 52.4 months, HR 0.72; 95% CI, 0.56–0.93) compared against the control arm [33]. During its recently published second interim analysis, the OS benefit significantly favored the pembrolizumab arm with 71% survival at 36 months compared with 64% in the placebo arm (HR 0.72; 95% CI 0.56–0.93; one-sided *P* = 0.0052) [34]. The EFS benefit was seen across all subgroups, regardless of PD-L1 expression, stage of disease, and NSCLC histology. Although the study did not exclude those with EGFR mutations or ALK translocations, no definitive conclusions can be drawn in this patient population due to a small sample size harboring these genomic drivers. A larger benefit in EFS was witnessed in stage III disease than stage II disease and in those who had an active or previous smoking history, although these observations could be due to smaller sample sizes. Those with squamous histology also derived significant benefit, which is meaningful in the context of a worse prognosis associated with squamous histology [35, 36]. Additionally, significantly increased major pathological response (30.2% vs. 11.0%) and pCR (18.1% vs. 4.0%) rates were observed in the pembrolizumab arm compared against the control arm, respectively [32]. There also appeared to be a positive correlation in the benefit derived from pembrolizumab and PD-L1 expression, although this was not statistically significant. Toxicities associated with treatment were increased in the pembrolizumab arm compared to the chemotherapy control arm. Grade 3–5 adverse events occurred in 44.9% and 37.3% of patients in the pembrolizumab and control arm, respectively, with chemotherapy associated adverse events such as anemia and nausea being among the most common events. Similarly, treatment-related adverse events which led to discontinuation of trial treatment occurred in 12.6% of patients in the pembrolizumab arm and in 5.3% of patients in the control arm. Immune-mediated adverse events occurred in 25.3% of patients in the pembrolizumab arm compared to 10.5% in the control arm. A higher rate of grade 3–5 immune-mediated adverse events was found in the pembrolizumab arm (7%) compared to the control arm (2%). The most frequently encountered grade 3 or higher immune-mediated adverse events in the pembrolizumab group included hypothyroidism, hyperthyroidism, severe skin reactions, and pneumonitis. Nevertheless, perioperative pembrolizumab subsequently gained FDA approval for patients with resectable NSCLC (tumors ≥ 4 cm or node positive) in combination with platinum-containing chemotherapy as neoadjuvant treatment, and then continued as a single agent as adjuvant treatment after surgery [37].

Shortly after reports of the KEYNOTE 671 data, results from the NADIM II trial, a randomized phase II study focusing exclusively on resectable stage IIIA or IIIB (AJCC 8th edition) NSCLC, was also reported. This study randomized 87 patients with resectable stage IIIA or IIIB NSCLC to carboplatin, paclitaxel, and nivolumab vs. carboplatin and paclitaxel prior to surgery, with an additional 6 months of adjuvant nivolumab in the experimental arm if R0 resection was achieved. The primary endpoint, pCR rates, following neoadjuvant chemoimmunotherapy vs. neoadjuvant chemotherapy, were 36% and 6.8%, respectively [38]. The study also met its secondary endpoints with a superior 24-month PFS (HR 0.47; 95% CI, 0.25 to 0.88) and OS benefit (HR 0.43; 95% CI, 0.19 to 0.98) observed with the addition of nivolumab. Of note, two-thirds of the patients enrolled in the study had pathologically proven N2 disease, including involvement of multiple N2 stations.

In another phase III, double-blind, placebo-controlled study, the AEGEAN trial, 802 patients with stage II–IIIB (N2 nodal disease) (AJCC 8th edition) NSCLC who were planned to undergo surgical resection (i.e., lobectomy, sleeve resection, or bilobectomy) were randomized to receive durvalumab or placebo every 3 weeks with four cycles of platinum-based chemotherapy prior to surgery, followed by durvalumab or placebo every 4 weeks for an additional 12 cycles after surgery [39]. Those with known EGFR or ALK alterations were excluded after protocol amendment. Primary endpoints were EFS and pCR. Durvalumab significantly improved EFS with a stratified HR of 0.68 (95% CI, 0.53–0.88) at the first interim analysis, and at the 1-year landmark analysis, the EFS was 73.4% in the chemo-ICI arm vs. 64.5% in the chemotherapy alone arm [39]. The pCR rate was also significantly higher with durvalumab at 17.2% vs. 4.3% in the chemotherapy alone arm. Improvements in EFS and pCR with durvalumab were witnessed across most subgroups. It is worth noting that although all stages derived statistically significant pCR benefit, the improvement in EFS was variable across disease stage; the benefit of durvalumab in stage IIIA disease appeared to be more prominent than those with stage II and IIIB disease. Additionally, the improvement in EFS and pCR from the addition of durvalumab in non-smokers appeared to be less pronounced. The greatest improvement in EFS and pCR seemed to be in previous or active smokers, consistent with other reports. No clear benefit was seen in the 51 patients harboring EGFR mutations. Adverse events leading to discontinuation of durvalumab or placebo occurred in 12% and 6% of patients, respectively. Grade 3 or 4 events related to treatment occurred in 32% of patients in the durvalumab arm and 32.9% of patients in the placebo group. Immune-mediated adverse events occurred in 23.7% of patients who received durvalumab and 9.3% of patients who received placebo, but only 4.2% of patients in the durvalumab arm experienced grade 3 or 4 immune-mediated events. Immune-mediated pneumonitis was reported in 3.7% of patients receiving durvalumab. The FDA subsequently granted approval for neoadjuvant durvalumab with platinum-containing chemotherapy followed by adjuvant durvalumab treatment in non-ALK/EGFR altered NSCLC with resectable tumors ≥ 4 cm and/or node involvement [40].

In the phase III Neotorch study conducted in China, 1,035 patients with stage II–IIIB (N2 nodal stage) (AJCC staging, 8th edition) NSCLC were treated with three cycles of platinum-based chemotherapy in combination with either toripalimab or placebo prior to surgery [41]. After surgery, patients received one more cycle of toripalimab or placebo in combination with chemotherapy, followed by maintenance toripalimab or placebo once every 3 weeks for up to 13 cycles. Patients with known EGFR or ALK alterations were excluded. EFS and major pathological response rates were the co-primary endpoints of the study. Results demonstrated an unreached EFS in the chemo-ICI arm vs. 15.1 months in the chemotherapy alone arm (HR 0.40; 95% CI, 0.28–0.5). Further, the major pathological response rate was 48.5% in the combination arm compared to 8.4% in the chemotherapy arm. The pCR rate was also higher in the combination arm (24.8% vs. 1.0%). The HR for median OS was 0.62 (95% CI, 0.38–1.00, *P* = 0.05). As observed in other studies, the benefit in EFS appeared to be more prominent in subgroups with PD-L1 ≥ 1% and in those with a positive smoking history. Notably, 78% of all enrolled patients had squamous NSCLC.

Following the CheckMate 816 study, the benefit of nivolumab was also evaluated in the perioperative setting in a randomized phase III trial, the CheckMate 77T [42]. This study randomized 461 patients with resectable stage IIA (> 4 cm) to IIIB (N2 node stage, single- or multistation) NSCLC (AJCC 8th edition) with no known EGFR mutations or ALK translocations to either neoadjuvant nivolumab or placebo with platinum-doublet chemotherapy for 4 cycles, followed by surgery and adjuvant nivolumab or placebo every 4 weeks for 1 year. The primary endpoint was EFS. At interim analysis, the 18-month EFS was 70.2% vs. 50.0% (HR 0.58; 97% CI, 0.42–0.81; *P* < 0.001) favoring the nivolumab arm. Similar to the observation from the CheckMate 816 study, a superior pCR rate (25.3% vs. 4.7%) was also achieved in the nivolumab arm. Treatment related adverse events that led to discontinuation of therapy occurred in 19.3% and 7.4% of patients in the nivolumab arm and chemotherapy group, respectively. The most common immune-mediated adverse events were thyroid disorders, which occurred in 11% of those in the nivolumab arm vs. 1.7% of those in the chemotherapy group. Following KEYNOTE 671 and AEGEAN studies, CheckMate 77T led to the FDA approval of a third peri-operative chemoimmunotherapy option in NSCLC with tumors ≥ 4 cm and/or node positivity without EGFR or ALK alterations [43].

Another PD-1 monoclonal antibody, tislelizumab, was also evaluated in the perioperative setting in a more recent phase III study, the RATIONALE-315 trial [44]. The study randomized 453 patients with stage II–IIIA NSCLC without EGFR mutations or ALK fusions to receive 3–4 cycles of tislelizumab or placebo in combination with platinum-based chemotherapy followed by surgery and eight cycles of adjuvant tislelizumab or placebo. Primary endpoints were major pathological response rate after the completion of neoadjuvant therapy and EFS. The median EFS and OS were not reached in either arm, but a statistically significant difference in EFS favoring tislelizumab has already been observed, with a HR of 0.56 (95% CI, 0.40–0.79; *P* = 0.0003).

To summarize, the benefit of chemo-ICI combination in the perioperative setting has been demonstrated in multiple randomized trials, and pembrolizumab-, durvalumab- and nivolumab-based regimens have gained FDA approval in stage II–IIIB resectable disease. Patients with resectable disease with tumor ≥ 4 cm and/or with node positive disease should be assessed for the peri-operative chemo-ICI combination as one of the mainstay treatment options.

### Immunotherapy strategies in the adjuvant setting

Following the establishment of adjuvant chemotherapy as the standard of care option in locally advanced NSCLC, evidence towards increased benefit from other adjuvant therapies in NSCLC were lacking for more than 15 years until the ADAURA trial, which reported improved outcomes with the adjuvant use of osimertinib in those with EGFR positive NSCLC [45]. Beyond this targeted treatment option, no other therapies had previously demonstrated added benefit in the adjuvant setting for resectable NSCLC. Efficacious signals from adjuvant ICI in other solid tumor subtypes, combined with proven efficacy of PD-L1 inhibition in the metastatic NSCLC setting, prompted investigation as to whether adjuvant use of ICI could provide further benefit to adjuvant chemotherapy. In the first phase III trial investigating the use of adjuvant immunotherapy after chemotherapy, the IMpower010 study compared adjuvant atezolizumab vs. best supportive care in completely resected (R0) stage IB–IIIA (AJCC 7th edition) patients after 1–4 cycles of cisplatin plus pemetrexed, gemcitabine, docetaxel, or vinorelbine [46]. The primary endpoint of DFS was assessed in multiple groups, including those with stage II–IIIA disease with PD-L1 ≥ 1%, all patients with stage II–IIIA disease, and all patients with stage IB–IIIA disease. Atezolizumab significantly increased DFS in the first and second groups with a HR of 0.66 (95% CI, 0.50–0.88) and 0.79 (95% CI, 0.64–0.96), respectively, but not in the third group. The EFS benefit was especially pronounced in those with a PD-L1 ≥ 50% (HR 0.43; 95% CI, 0.27–0.68). A positive trend in OS was also observed with the use of atezolizumab in stage II–IIIA with positive PD-L1 expression, likely driven by the benefit witnessed in those with the PD-L1 ≥ 50% [47]. Toxicities were naturally increased in the atezolizumab arm compared to best supportive care; grade 3–5 events occurred in 24% and 13% of patients in the atezolizumab and supportive care arm, respectively. The most common grade 3 or 4 adverse events in the atezolizumab group were increased alanine aminotransferase and aspartate aminotransferase levels and pneumonia. Atezolizumab discontinuation due to adverse events occurred in 18% of patients, most frequently because of hypothyroidism, pneumonitis, and increased aspartate aminotransferase. Immune-related adverse events occurred in 52% of patients in the atezolizumab arm compared to 9% in the best supportive care arm. Of these events, 8% of patients in the atezolizumab group experienced grade 3 or 4 events compared to 1% in the placebo arm. The most common atezolizumab-related adverse events were hypothyroidism (11%), pruritis (9%), and rash (8%). Grade 5 atezolizumab-related adverse events occurred in four patients. Treatment with corticosteroids was required in 12% of patients treated with atezolizumab for immune-mediated adverse events. The FDA approved the use of adjuvant atezolizumab in stage II to IIIA NSCLC with PD-L1 expression ≥ 1% on tumor cells, whereas NCCN guidelines support the use of adjuvant atezolizumab in stage IIB–IIIA, stage IIIB (T3, N2), or high-risk stage IIA NSCLC with PD-L1 ≥ 1% [6]. As discussed previously, NSCLC with EGFR or ALK alterations should be excluded from this approach.

Following the IMpower010 study, the PEARLS/KEYNOTE 091 study investigated the use of adjuvant pembrolizumab in stage IB–IIIA (AJCC 7th edition) NSCLC patients following consideration of adjuvant chemotherapy [48]. The study had dual primary endpoints of DFS both in the overall population and in those with a PD-L1 TPS ≥ 50%. The study only met its endpoint in the PD-L1-unselected patient population with a HR of 0.76 (95% CI, 0.63–0.91, *P* = 0.0014) favoring pembrolizumab, while unexpectedly, no statistically significant benefit was witnessed in patients with a PD-L1 TPS ≥ 50%. The reason for this negative finding in those with a PD-L1 ≥ 50% remains unclear but may be attributable to over performance in the placebo group due to an imbalance of unknown factors. The benefit in the PD-L1 TPS < 1% population was also less clear in the subgroup analysis. Survival outcomes have not yet been reported. Adverse events leading to discontinuation of treatment occurred in 20% of those receiving pembrolizumab vs. 6% on placebo. Adverse events directly attributed to pembrolizumab which led to treatment discontinuation occurred in 17% of patients, with the most frequent events being hypothyroidism (20%), pruritus (18%), diarrhea (13%), and fatigue (11%). Grade 3 or worse immune-mediated adverse events which occurred in the pembrolizumab arm included 5 patients and included severe skin reactions, hepatitis, and pneumonitis. Two patients died presumably due to immune-mediated myocarditis secondary to pembrolizumab exposure. Adjuvant pembrolizumab gained FDA approval, irrespective of PD-L1 score, in stage IB (≥ 4 cm), II and IIIA NSCLC, although NCCN guidelines recommended this approach be offered in stage IIB–IIIA, stage IIIB (T3, N2), or high-risk stage IIA (AJCC 8th edition) NSCLC [6].

In another more recent phase III study, the ADJUVANT BR.31 trial, adjuvant administration of durvalumab was assessed in 1,415 patients [49]. When compared to placebo, adjuvant durvalumab did not lead to significant DFS in stage IB (≥ 4 cm)-IIIA NSCLC in those with a PD-L1 expression of 25% or more following surgical resection. More detailed data and results are necessary to understand potential causes for these negative findings.

In summary, while both adjuvant atezolizumab and pembrolizumab can be offered to non-ALK and non-EGFR altered NSCLC patients following adjuvant chemotherapy, PD-L1 ≥ 1% is required for atezolizumab use while the administration of pembrolizumab is irrespective of PD-L1 score. Patients with ALK rearrangements or EGFR mutations are unlikely to derive benefit from PD-1/PD-L1 inhibition, and therefore, should be excluded from consideration of immunotherapy [29, 30]. Instead, patients with EGFR mutations and ALK rearrangements should receive adjuvant osimertinib or alectinib, respectively, based on the results from the ADAURA and ALINA trials [50, 51].

A summary of the major phase III trials describing the various FDA approved chemo-ICI combinations in the neoadjuvant, perioperative, and adjuvant settings can be found in Table 1. In addition to these regimens, several additional PD-L1 inhibitors are being evaluated in phase III trials with similar designs. Some of these ongoing trials are utilizing predictive platforms such as minimal residual disease (MRD) detection or pCR status to help guide adjuvant treatment decision making in an attempt to identify which patients are most likely to derive benefit from post-operative adjuvant treatment or require immunotherapy intensification. These trials will hopefully help delineate the contribution and benefit of adjuvant ICI in the perioperative chemo-ICI realm (Table 2).

**Table 1:** Summary of phase III trials in the neoadjuvant, perioperative, and adjuvant settings that led to FDA approval

Trial (NCT#)	Stage (AJCC); patient traits	Regimen	Primary endpoint results	FDA approval indication
IMpower010 (NCT02486718)	IB (≥ 4 cm)–IIIA (7th)	Adjuvant chemo followed by atezolizumab every 3 weeks for up to 16 cycles	DFS in stage II–IIIA (PD-L1 ≥ 1%), all stage II–IIIA and ITT populations: HR 0.66 (*P* = 0.0039); 0.79 (*P* = 0·020) and 0.81 (*P* = 0.040)	Adjuvant treatment following resection and platinum-based chemotherapy for adult patients with stage II to IIIA NSCLC whose tumors have PD-L1 expression on ≥ 1% of tumor cells, as determined by an FDA-approved test
KEYNOTE 091/PEARLS (NCT02504372)	IB (≥ 4 cm)–IIIA (7th)	Adjuvant chemo followed by pembrolizumab every 3 weeks for up to 18 cycles	DFS in all population and PD-L1 TPS ≥ 50%: HR 0.76, *P* = 0.0014 and HR 0.82, *P* = 0.14	Adjuvant treatment following resection and platinum-based chemotherapy for adult patients with stage IB (T2a ≥ 4 cm), II, or IIIA disease
CheckMate 816 (NCT02998528)	IB (≥ 4 cm)–IIIA (7th); irrespective of PD-L1	Neoadjuvant nivolumab plus chemo every 3 weeks for 3 cycles	EFS: HR 0.63, *P* = 0.005 pCR: 24.0% vs. 2.2% Odds ratio 13.9, *P* < 0.001	Resectable disease (tumors ≥ 4 cm or node positive); in combination with platinum-doublet chemotherapy in the neoadjuvant setting
KEYNOTE 671 (NCT03425643)	II–IIIB (N2 stage) (8th)	Pembrolizumab plus chemo × 4 cycles; surgery; adjuvant pembrolizumab every 3 weeks for up to 13 cycles	EFS: HR 0.58, *P* < 0.00001 OS: HR 0.72, *P* = 0.00517	Resectable disease (tumors ≥ 4 cm or node positive); in combination with platinum-containing chemotherapy as neoadjuvant treatment, and then continued as a single agent as adjuvant treatment after surgery
AEGEAN (NCT03800134)	IIA to stage IIIB (N2 stage) (8th)	Durvalumab plus chemo × 4 cycles; surgery; adjuvant durvalumab every 4 weeks for up to 12 cycles	EFS: HR 0.68, *P* = 0.0004 pCR: 17.2% vs. 4.3%, *P* < 0.001	Resectable (tumors ≥ 4 cm and/or node positive) with no known EGFR or ALK alterations; in combination with platinum-containing chemotherapy as neoadjuvant treatment, and then continued as a single agent as adjuvant treatment after surgery
CheckMate 77T (NCT04025879)	IIA to stage IIIB (single- or multistation N2) (8th)	Nivolumab plus chemo × 4 cycles; surgery; adjuvant nivolumab every 4 weeks for one year	EFS: HR 0.58, *P* < 0.001 pCR: 25.3% vs. 4.7% Odds ratio, 6.64; 95% CI, 3.40 to 12.97 Major pathological response: 35.4% vs. 12.1% Odds ratio, 4.01; 95% CI, 2.48 to 6.49	Resectable (tumors ≥ 4 cm and/or node positive) with no known EGFR or ALK alterations; in combination with platinum-containing chemotherapy as neoadjuvant treatment, and then continued as a single agent as adjuvant treatment after surgery

EGFR: epidermal growth factor receptor; HR: hazard ratio; EFS: event free survival; FDA: Food and Drug Administration; ITT: intention-to-treat; NSCLC: non-small cell lung cancer; OS: overall survival; pCR: pathological complete response

**Table 2:** Selected ongoing phase III trials evaluating neoadjuvant, perioperative, and adjuvant chemoimmunotherapy in resectable NSCLC

**Trial**	**Patient population**	**Treatment**	**Primary endpoint**	**Study completion**
MERMAID-1 (NCT04385368)	Stage II/III NSCLC after resection	Adjuvant durvalumab or placebo plus chemotherapy for 12 weeks followed by durvalumab or placebo for up to week 48	DFS in the MRD+ (measured by whole exome sequencing-based ctDNA test) group	2023-8-31
ANVIL (NCT02595944)	Stage IB (≥ 4 cm)–IIIA NSCLC after resection	Adjuvant nivolumab or observation	OS and DFS	2025-12-31
LungMate-008 (NCT04772287)	Stage II–IIIB (N2) NSCLC after resection	Adjuvant toripalimab or placebo with chemotherapy followed by 4 cycles adjuvant toripalimab or placebo	DFS	2027-12-30
ADOPT-lung (NCT06284317)	Resectable IIB–IIIB (N2)	Adjuvant durvalumab for 12 cycles or observation	DFS	2030-03
NADIM-ADJUVANT (NCT04564157)	Stage IB–IIIA NSCLC after resection	Adjuvant nivolumab with chemotherapy followed by 6-month of nivolumab vs. adjuvant chemotherapy	DFS	2031-04-01
IMpower 030 (NCT03456063)	Resectable stage II, IIIA, or select IIIB	Four cycles of neoadjuvant atezolizumab with chemotherapy and up to 16 cycles of adjuvant atezolizumab or placebo with chemotherapy	EFS	2025-01-19
PROSPECT LUNG (NCT06632327)	Resectable stage IIA to IIIB	Neoadjuvant ICI with 4 cycles of chemotherapy and one year adjuvant ICI vs. adjuvant therapy with 4 cycles chemotherapy and one year ICI	3-year real-world event-free survival (rwEFS) and OS	2030-04-30
CLEAR-INSIGHT (INSIGHT: NCT06498635)	Stage II–IIIB after resection	SOC neoadjuvant platinum-based chemotherapy and anti-PD-(L)1 therapy then surgery pCR: adjuvant durvalumab for 12 cycles vs. observation (INSIGHT) Non-pCR: adjuvant durvalumab and novel inhibitor combination vs. durvalumab for one year	DFS (INSIGHT)	2039-07-15 (INSIGHT)

MRD: minimal residual disease; PD-1: programmed death 1; PD-L1: programmed death 1 ligand; ctDNA: circulating tumor DNA; EFS: event free survival; ICI: immune checkpoint inhibitors; NSCLC: non-small cell lung cancer; OS: overall survival; pCR: pathological complete response

### Treatment selection for patients with resectable stage II and III NSCLC and unmet needs

As summarized above, six treatment regimens employing three different strategies, including neoadjuvant treatment only, adjuvant treatment only, and peri-operative treatment, have been approved by the FDA in patients with resectable stage II and III NSCLC without ALK/EGFR alterations. However, due to the absence of head-to-head trials, the contribution of PD-(L)1 inhibitors in the neoadjuvant and the adjuvant phases of treatment have not been fully characterized, and there is no definitive data to guide treatment selection among these options. Nevertheless, the perioperative pembrolizumab regimen is the only one that has demonstrated significant OS benefit, whereas the significance boundary for OS in the neoadjuvant CheckMate 816 study still has not been met at its most recent 4-year updated analysis, placing the KEYNOTE 671 regimen in a more favorable position when compared with the other options. The difference in the EFS HR in patients who did not achieve pCR when comparing the neoadjuvant and peri-operative approaches in their respective trials also suggested increased benefit with the perioperative approach. In both the neoadjuvant and perioperative trials, the patients who did not achieve pCR had shorter EFS. However, in two of the three peri-operative treatment trials, significantly improved EFS was observed with the addition of ICI even in this subset of patients; the HR for EFS from the KEYNOTE 671, Neotorch, and CheckMate 77T trials were 0.69 (95% CI, 0.55–0.85), 0.53 (95% CI, 0.38–0.74) and 0.70 (95% CI, 0.43–1.13), respectively [32, 41, 42]. Conversely, in the CheckMate 816, although there was a trend favoring neoadjuvant nivolumab, a higher EFS HR of 0.84 (95% CI, 0.61–1.17) was observed among patients without a pCR, suggesting that three doses of ICI may not be sufficient and a longer exposure to ICI may be required for those who do not achieve pCR.

Toxicities and tolerability should also be a significant factor when considering treatment options, recognizing the chance for increased toxicities with one-year of adjuvant ICI treatment (Table 3). In a metanalysis comparing rates of ICI-mediated toxicity among 15 phase III clinical trials that have led to FDA approval of ICIs in the neoadjuvant, adjuvant, peri-operative, and palliative settings, the neoadjuvant-only approach was associated with the lowest rates of immune-mediated adverse events. Since side effects could negatively impact quality of the life (QoL), QoL was also included as a secondary endpoint in the perioperative trials of KEYNOTE 671, CheckMate 77T, and AEGEAN [34, 39, 52]. Data from KEYNOTE 671 showed no long-term decrease in health-related QoL associated with pembrolizumab use when compared with placebo. Although health-related QoL scores initially decreased during the neoadjuvant phase, they subsequently returned to near baseline levels during the adjuvant phase in both the KEYNOTE 671 and CheckMate 77T trials. Moreover, a significantly longer time until definitive deterioration in disease-related symptoms was observed in patients in the nivolumab arm compared with the placebo arm (HR 0.66; 95% CI, 0.45 to 0.98) in the CheckMate 77T, suggesting that there is improved QoL associated with improved disease control [42]. Quality of life data from the AEGEAN study has not yet been published. In short, although immune-mediated toxicities are generally increased in the perioperative approach, the QoL data is reassuring when choosing to offer the perioperative treatment option to patients.

**Table 3:** Toxicities and surgery completion rates in patients underwent treatments with FDA-approved neoadjuvant, adjuvant and perioperative regimens

Trial (NCT#)	Adverse events (ICI vs. placebo)				Patients who completed surgery (ICI vs. placebo)
	**Total**	**Neoadjuvant**	**Adjuvant**	**ICI mediated**	
IMpower010 (NCT02486718)	93% vs. 71%	N/A	93% vs. 71%	52% vs. 9%	100% vs. 100%
KEYNOTE 091/PEARLS (NCT02504372)	96% vs. 91%	N/A	96% vs. 91%	39% vs. 13%	100% vs. 100%
CheckMate 816 (NCT02998528)	92.6% vs. 97.2%	92.6% vs. 97.2%	N/A	20% vs. 1%	83.2% vs. 75.4%
KEYNOTE 671 (NCT03425643)	96.7% vs. 95%	95.7% vs. 93.7% (neoadjuvant + surgery phase)	54.5% vs. 31.8%	25.3% vs. 10.5%	82.1% vs. 79.4%
AEGEAN (NCT03800134)	96.5% vs. 94.7%	91.0% vs. 89.2%	Unknown	23.7% vs. 9.3%	77.6% vs. 76.7%
CheckMate 77T (NCT04025879)	97.4% vs. 97.8%	94.7% vs. 96.1%	87.3% vs. 79.6%	35.2% vs. 7.8%	77.7% vs. 76.7%

FDA: Food and Drug Administration; ICI: immune checkpoint inhibitors

**Figure 3:**
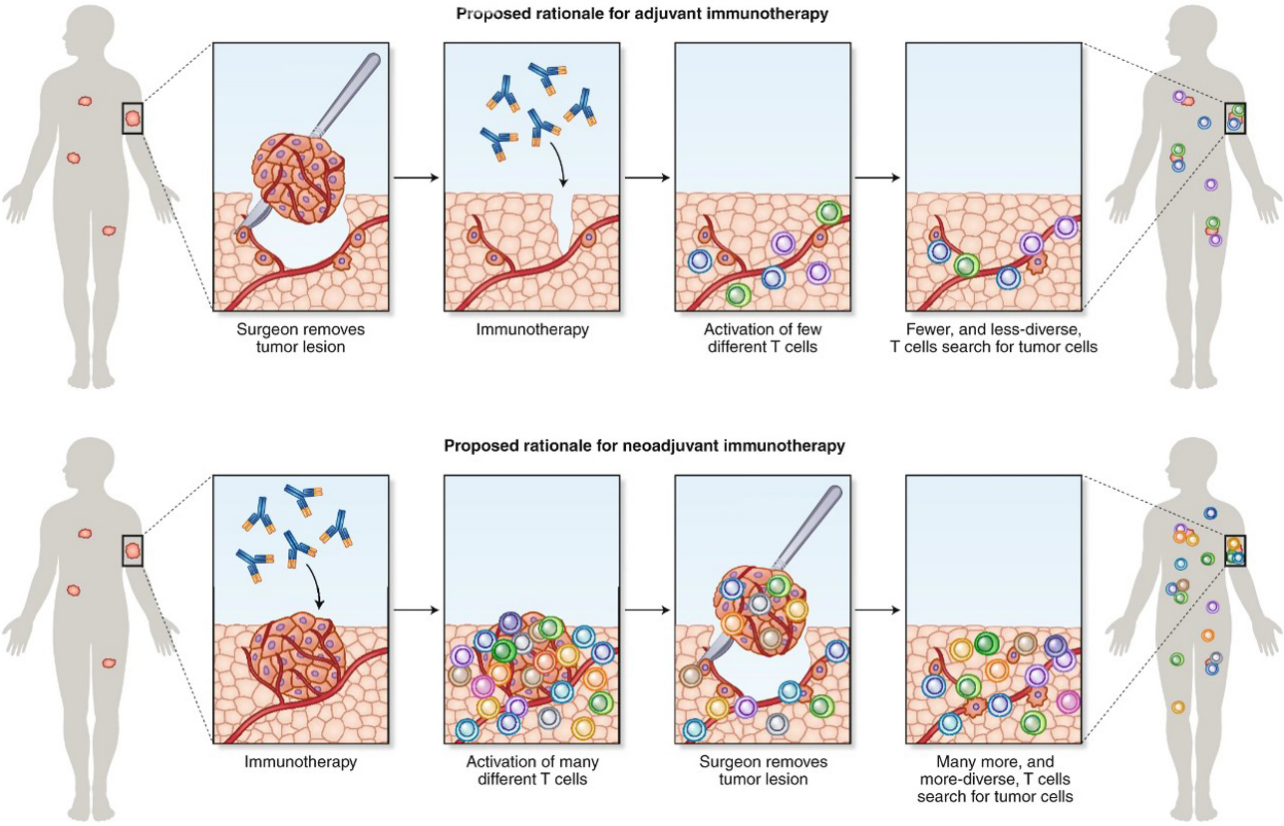
Rationale for neoadjuvant and adjuvant administration of immunotherapy [55] *Note.* Adapted with permission from “Learning from clinical trials of neoadjuvant checkpoint blockade” by Versluis JM, Long GV, Blank CU. Nat Med. 2020;26:475–84 (https://www.nature.com/articles/s41591-020-0829-0). Copyright © 2020, Springer Nature America, Inc

When compared with the adjuvant treatment approach, from a mechanistic standpoint, the pharmacological inhibition of PD-1 and/or PD-L1 in the neoadjuvant setting may have the advantage of evoking a stronger signal for host T-cells to attack tumor cells due to larger tumor antigen burden prior to surgical resection of the tumor and removal of tumor-infiltrating lymphocytes [53, 54] (Figure 3). While the perioperative treatment option may be the preferred option for many patients, it is worth noting that the adjuvant treatment approach has the additional advantage of higher surgery completion rates. Around 20% of the patients in the neoadjuvant only or perioperative treatment trials were unable to complete surgery, the definitive treatment modality, due to various reasons, including treatment related adverse events, poor compliance, and disease progression. The adjuvant only treatment approach also has the benefit of being able to provide enough tumor tissue for molecular profiling, which is required before neoadjuvant chemo-ICI use so as to exclude those with ALK/EGFR alterations. Therefore, for patients who are at high risk of missing surgery or who might have insufficient tissue for ALK/EFGR status evaluation, especially in those with a high likelihood of having these alterations, the adjuvant only treatment approach would be the preferred option. Additionally, in patients who are upstaged to stage II or above after upfront surgery, adjuvant ICI would be indicated after completion of adjuvant chemotherapy.

The optimal duration of ICI has not been determined in either the early stage or recurrent/metastatic NSCLC. Moreover, identifying which patients might benefit from adjuvant phases of treatment remains a challenge. The excellent EFS witnessed in those who achieve pCR after surgical resection begs the question as to how to identify which patients can omit adjuvant therapy. The negative results in the ADJUVANT BR.31 trial presents conflicting evidence towards the contribution of adjuvant durvalumab when compared with the perioperative AEGEAN trial, emphasizing the need for newer trial designs in order to address the contribution of benefit from each distinct phase of treatment. The FDA’s Oncologic Advisory Drugs Committee meeting from July 25, 2024, recently highlighted this need to address the contribution and value of each treatment phase as they encouraged future trial designs which would allow for a new therapy to be added to either the neoadjuvant or adjuvant phase alone (i.e., multi-arm trials, factorial trials, trials that allow for re-randomization etc.) [56].While ongoing trials such as PROSPECT-LUNG (NCT 06632327) and INSIGHT (NCT 06498635) are seeking to directly compare perioperative and neoadjuvant treatment regimens, these trials unfortunately will still be unable to delineate the precise contribution of the neoadjuvant phase of treatment due to its comparison with a perioperative approach The primary challenge of conducting a multi-arm study is the large sample size and time that is required for such a trial completion, especially if OS is included as a primary endpoint. Additionally, due to the rapidly evolving landscape in NSCLC, the standard of care options utilized in such time-consuming trials may change before trial completion. While awaiting more informative data regarding the value of each phase of treatment, the positive correlation between pCR and EFS should be shared during treatment plan discussions with patients in order to help them make an informed decision based on their preferences and priorities.

Among patients who did receive adjuvant ICI, some still developed disease progression despite adequate exposure to PD-(L)1 inhibitors, suggesting that treatment strategies other than simply continuing the same ICI should be employed to overcome the primary resistance to PD-(L)1 inhibitors in this subgroup of patients. How to identify diseases with primary resistance to immunotherapy posts a significant challenge. PD-L1 expression and tumor mutation burden (TMB) are the only currently available biomarkers for ICI efficacy, but their predictive power has been disappointing. In a meta-analysis of 3,387 patients from eight phase 2 or 3 randomized control studies evaluating neoadjuvant chemo-ICI with or without adjuvant ICIs, a significant 2-year EFS benefit was observed in both PD-L1 positive and PD-L1 negative subgroups [57]. Various tumor intrinsic and extrinsic factors contributing to resistance mechanisms have been identified, such as lack of tumor immunogenicity due to low TMB, neoantigen loss, lack of antigen presentations, genetic T cell exclusion, insensitivity to T-cells, absence of T-cells in the tumor microenvironment, inhibitory immune checkpoints, and immunosuppressive cells [58]. In NSCLC, numerous types of biomarkers predicting intrinsic and extrinsic resistance have been investigated, including gene-expression, peripheral blood mononuclear cells, tumor-infiltrating immune cells, extracellular vesicles, gut microbial signatures, integrated models incorporating TMB adjusted for tumor purity, activating mutations in receptor tyrosine kinase mutations, molecular smoking signature, and human leukocyte antigen status [59–61]. Notably, artificial intelligence-based analyses of tumor images has also been evaluated as a possible predictive biomarker and radiomic signatures have been identified to possibly predict immunotherapy efficacy [62, 63]. These biomarkers still need to be increasingly studied and validated in patients undergoing neoadjuvant, adjuvant, or perioperative treatment before being able to aid in the development of treatment algorithms so as to provide individualized, patient-tailored, treatment strategies. Novel immunogenic agents also remain a possibility towards future trial designs. One example includes ivonescimab, a bispecific antibody against PD-1 and VEGF, which demonstrated significantly improved PFS when compared with pembrolizumab in treatment naïve stage IV NSCLC in those with a PD-L1 TPS ≥ 1% [64]. Agents such as these maybe considered for incorporation into future clinical trials in the perioperative realm, particularly for patients who do not achieve pCR.

## The role of immunotherapy in NSCLC undergoing curative-intent radiation

### Immunotherapy in stage I–II NSCLC treated with stereotactic body radiotherapy (SBRT)

Stereotactic body radiotherapy (SBRT) or stereotactic ablative body radiotherapy (SABR) is the standard treatment for patients with medically inoperable early-stage NSCLC or in those who refuse surgery. While local control can be achieved in more than 90% of patients in the irradiated field, regional or distant recurrences may occur in as many as 11–20% patients [65–67]. In an effort to improve the outcome of these patients, a phase 2 randomized trial compared SABR with or without 16 weeks (4 doses) of nivolumab in patients with primary or isolated lung parenchymal recurrent tumor of 7 cm or less with no active nodal disease who were unable or unwilling to undergo surgery. The study met its primary endpoint and showed an improved 4-year EFS with a HR of 0.38 (95% CI 0.19–0.75) [67]. However, this benefit from immunotherapy was not witnessed in a separate phase 3 trial, KEYNOTE 867, which compared SBRT followed by one year of pembrolizumab or placebo in patients with stage I–II NSCLC with no nodal involvement who were unable or unwilling to undergo surgery. The trial unfortunately missed its primary endpoint of recurrence-free survival and the study was discontinued [68]. Once made available to public, the data from this study will hopefully help provide clearer insights into the benefits of immunotherapy according to various subgroups, its impact on local regional vs. distant recurrence, as well as its safety profile, so as to help guide future trial design.

### Strategies in unresectable stage III disease

Unresectable disease generally includes some stage IIIA tumors, most stage IIIB tumors, and all patients with stage IIIC disease. Although determining resectability status in stage IIIA disease remains a specific area of controversy and is best decided in multidisciplinary fashion, NCCN guidelines help direct decision making in this difficult realm based primarily on the number and size of N2 disease [6]. Following these determinations, patients with diseases that are deemed as unresectable, or who are not surgical candidates, are treated with definitive CRT as the standard upfront treatment approach with few exceptions [69–72]. There was formerly no established role for immunotherapy in this setting until preliminary studies began to discover upregulation of PD-L1 expression on tumor cells following the administration of chemotherapy and radiation treatments [73, 74]. Subsequently, the landmark PACIFIC study was the first phase III, randomized, placebo-controlled, trial to investigate and compare the role of durvalumab against placebo as consolidative therapy following CRT in stage III (AJCC 7th edition) unresectable disease [75]. Patients were assigned to durvalumab or placebo every 2 weeks for up to 1 year following completion of CRT. The primary endpoints were PFS and OS. The study met both endpoints; the HR for OS and PFS were 0.71 and 0.55, respectively [76]. The post-hoc analysis revealed significantly improved PFS and OS in all subgroups except in those with a PD-L1 ≤ 1%. Treatment related adverse events occurred in 67.8% of patients in the durvalumab arm compared to 53.4% of patients in the placebo group. Grade 3 or 4 adverse events occurred in 11.8% and 4.3% of patients in the durvalumab and placebo arms, respectively. Of those adverse events which were immune mediated, grade 3 or higher adverse events occurred in 3.4% of patients in the durvalumab arm and included pneumonitis (1.7%), rash (0.4%), and hypothyroidism (0.2%). In short, the trial clearly established consolidative durvalumab as the standard of care treatment option in stage III disease following completion of definitive CRT and gained FDA approval in this setting. NCCN guidelines recommend durvalumab in stage III (category 1) and stage II (category 2A) NSCLC following CRT [6]. It is worth noting that those with EGFR mutated NSCLC should not be offered consolidative durvalumab following CRT completion. Instead, in this setting, adjuvant osimertinib is recommended based on results from the recent phase 3 LAURA study [77].

Although durvalumab following concurrent CRT significantly improved OS in those with unresectable stage III disease, outcomes in this subset of patients remains to be improved. Since close to 30% of patients in the PACIFIC study were ineligible to receive durvalumab due to disease progression or significant toxicity from CRT [75], two randomized phase III studies, PACIFIC-2, and CheckMate 73L, evaluated the benefit of adding ICI to concurrent CRT. Unfortunately, both studies failed to meet their primary endpoints of PFS and no benefit was detected when compared with the standard of care control arm [78, 79]. Immunotherapy intensification by combining PD-(L)1 inhibitor with other immunotherapy agents such as TIGIT, CD73, NKG2A receptor, or CTLA-4 monoclonal antibodies, or targeted therapy agents such as PARP inhibitors or multikinase inhibitors, is being evaluated in ongoing trials. Given the added toxicity and financial burden associated with treatment escalation, investigators are attempting to incorporate predictive biomarkers such as post-CRT ctDNA within study designs in order to personalize consolidative immunotherapy based on one’s risks of recurrence. PD-L1 expression-driven enrichment strategies are also being used in some studies to maximize the benefit of immunotherapy. The ongoing phase III clinical trials investigating the role of immunotherapy in unresectable disease are summarized in Table 4.

**Table 4:** Selected ongoing phase III trials utilizing immunotherapy in unresectable disease

**Trial**	**Stage (AJCC 8th edition)**	**Treatment**	**Primary endpoint**
KEYLYNK-012 (NCT04380636)	Stage IIIA–C	Pembrolizumab plus CRT followed by pembrolizumab with/without olaparib vs. CRT followed by durvalumab	PFS and OS
EA5181 (NCT04092283)	Stage III	Durvalumab plus CRT or CRT, followed by one year of durvalumab	OS
Skyscraper-03 (NCT04513925)	Stage III after CRT	Atezolizumab plus tiragolumab or durvalumab	PFS
Pacific-9 (NCT05221840)	Stage III after CRT	Durvalumab plus oleclumab or monalizumab or placebo	PFS
NCT04325763	Stage III after CRT	TQB2450 plus anlotinib or TQB2450 or placebo	PFS
Pacific-8 (NCT05211895)	PD-L1 positive stage III after CRT	Durvalumab plus either domvanalimab or placebo	PFS
NCT04585490	Stage III after CRT	MRD negative patients after CRT will receive consolidation durvalumab and MRD-positive patients will receive durvalumab plus four additional cycles of tremelimumab chemotherapy	MRD change as measured by ctDNA after tremelimumab chemotherapy

MRD: minimal residual disease; PD-L1: programmed death 1 ligand; CRT: chemoradiation; ctDNA: circulating tumor DNA; OS: overall survival; PFS: progression-free survival

## Conclusions

The substantial benefits and tremendous progress made from the inclusion of immunotherapy in locally advanced NSCLC have been demonstrated in multiple phase III randomized studies in the curative-intent treatment approach, including neoadjuvant, perioperative, and adjuvant chemo-ICI combination therapies in those with surgically resectable disease. Curative intent use of immunotherapy in those with unresectable disease (or poor surgical candidacy) has also been demonstrated significant benefit following treatment with definitive CRT. However, more data is necessary in order to provide further expansive insights to help guide the personalized decision-making process with patients. In the perioperative realm, further randomized trials are also needed to delineate the role and additive value (or lack thereof) associated with each phase of treatment in those with resectable disease. Predictive biomarkers will be critical in this regard to help elucidate which patients have diseases with primary resistance to ICI and may benefit from treatment intensification with the addition of novel therapeutic agents. Moreover, there have been no prospective trials comparing CRT followed by consolidative immunotherapy against surgical approaches involving immunotherapy regimens (i.e., neoadjuvant or perioperative immunotherapy). Until such data becomes available, and given the complexity and variability across disease presentation, as well as the multiple available treatment options based on patient-specific characteristics, it is worth emphasizing that treatment decisions always be made in a multidisciplinary fashion and through shared decision making with the patient.

## Abbreviations

Chemo-ICI: chemotherapy-immune checkpoint inhibitor
CRT: chemoradiation
ctDNA: circulating tumor DNA
CTLA-4: cytotoxic T lymphocyte antigen 4
EFS: event free survival
EGFR: epidermal growth factor receptor
FDA: Food and Drug Administration
HR: hazard ratio
ICI: immune checkpoint inhibitors
ITT: intention-to-treat
MRD: minimal residual disease
NSCLC: non-small cell lung cancer
OS: overall survival
pCR: pathological complete response
PD-1: programmed death 1
PD-L1: programmed death 1 ligand
PFS: progression-free survival
QoL: quality of the life
SABR: stereotactic ablative body radiotherapy
SBRT: stereotactic body radiotherapy

## Declarations

### Copyright

© The author(s) 2024.
